# Effects of the on-site energy on the electronic response of Sr_3_(Ir_1−*x*_Mn_*x*_)_2_O_7_

**DOI:** 10.1038/s41598-022-23593-z

**Published:** 2022-11-08

**Authors:** Dongwook Kim, G. Ahn, J. Schmehr, S. D. Wilson, S. J. Moon

**Affiliations:** 1 Department of Physics, Hanyang University, Seoul, 04763 Republic of Korea; 2 Materials Department, University of California, Santa Barbara, CA 93106 USA; 3 Institute for High Pressure, Hanyang University, Seoul, 04763 Republic of Korea; 4 Research Institute for Natural Sciences, Hanyang University, Seoul, 04763, Republic of Korea

**Keywords:** Condensed-matter physics, Electronic properties and materials

## Abstract

We investigated the doping and temperature evolutions of the optical response of Sr_3_(Ir_1−*x*_Mn_*x*_)_2_O_7_ single crystals with 0 ≤ *x* ≤ 0.36 by utilizing infrared spectroscopy. Substitution of 3*d* transition metal Mn ions into Sr_3_Ir_2_O_7_ is expected to induce an insulator-to-metal transition via the decrease in the magnitude of the spin–orbit coupling and the hole doping. In sharp contrast, our data reveal the resilience of the spin–orbit coupling and the incoherent character of the charge transport. Upon Mn substitution, an incoherent in-gap excitation at about 0.25 eV appeared with the decrease in the strength of the optical transitions between the effective total angular momentum *J*_eff_ bands of the Ir ions. The resonance energies of the optical transitions between the *J*_eff_ bands which are directly proportional to the magnitude of the spin–orbit coupling hardly varied. In addition to these evolutions of the low-energy response, Mn substitution led to the emergence of a distinct high-energy optical excitation at about 1.2 eV which is larger than the resonance energies of the optical transitions between the *J*_eff_ bands. This observation indicates that the Mn 3*d* states are located away from the Ir 5*d* states in energy and that the large difference in the on-site energies of the transition metal ions is responsible for the incoherent charge transport and the robustness of the spin–orbit coupling. The effect of Mn substitution was also registered in the temperature dependence of the electronic response. The anomaly in the optical response of the parent compound observed at the antiferromagnetic transition temperature is notably suppressed in the Mn-doped compounds despite the persistence of the long-range antiferromagnetic ordering. The suppression of the spin-charge coupling could be related to charge disproportionation of the Ir ions.

## Introduction

Layered perovskite iridates of the Sr_n+1_Ir_n_O_3n+1_ (n = 1, 2) have attracted a great deal of attention as candidate systems from which unconventional superconductivity may emerge^1–4^. Sr_n+1_Ir_n_O_3n+1_ is an effective total angular momentum *J*_eff_ = 1/2 Mott insulator realized by the cooperation of the moderate electronic correlations and strong spin–orbit coupling (SOC)^5–7^. The *J*_eff_ = 1/2 Mott state of the iridates shares close similarities in its electromagnetic properties with the cuprates, which motivated intensive efforts to search for novel phases of the iridates by means of charge carrier doping. The representative cuprate phenomenology including the pseudogap^8,9^, the *d*-wave gap^9,10^, charge density wave^11^ was also observed in doped iridates.

Since the Mott state of the iridates is stabilized by the strong SOC, it is expected that the reduction of the SOC may lead to an insulator-metal transition and associated novel phases. Theoretical studies suggested that the singlet *d*-wave and triplet *p*-wave pairing state could appear with the control of SOC^2,4^. The magnitude of the relativistic SOC is known to be proportional to the Z^2^ (Z: atomic number)^12^, thus it can be controlled via substitution of Ir ions with other transition metal ions. A recent angle-resolved photoemission spectroscopy (ARPES) study on Sr_2_(Ir_1−*x*_Rh_*x*_)O_4_ and Sr_2_(Ir_1−*x*_Ru_*x*_)O_4_ suggested that the Rh/Ru doping resulted in the reduction of the SOC via hybridization between doped Rh/Ru ions and the host Ir ions, and this SOC reduction played a decisive role for their insulator-metal transition^13^. It was also suggested that the efficiency in the SOC dilution depended on the closeness of the on-site energies of the states of the Ir and Rh/Ru ions. On the other hand, another ARPES measurement indicated that the SOC did not control the insulator-metal transition of Sr_2_(Ir_1−*x*_Rh_*x*_)O_4_ and Sr_2_(Ir_1−*x*_Ru_*x*_)O_4_. Instead, this study showed that the rigid band shift by hole doping and the appearance of the sets of bands with mostly Ru character generated by the hybridization between Ru and Ir ions drove the insulator-metal transition in Sr_2_(Ir_1−*x*_Rh_*x*_)O_4_ and Sr_2_(Ir_1−*x*_Ru_*x*_)O_4_, respectively^14^. The distinct origins of the insulator-metal transitions in the two systems were attributed to the difference in the on-site energies of the Rh and Ru ions.

Doping of 3*d* transition metal ions may be more effective in studying the effects of the change in the SOC to the electronic response of the iridates and the roles of the on-site energy of the host and impurity ions for the SOC dilution. Several studies investigated the transport and magnetic properties of Fe- or Co-doped Sr_2_IrO_4_, which showed that Fe or Co doping induced insulator-metal transitions^15,16^. The insulator-metal transition was attributed to the formation of the impurity states close to the Fermi level. The effects of the Fe/Co doping on the electronic structure and the SOC, which can be gained by spectroscopy studies, were not discussed in these reports^15,16^. To the best of our knowledge, there is no optical spectroscopy reported on the 3*d* transition-metal-doped iridates.

In this paper, we studied the doping and temperature evolutions of the electronic response of Mn-doped Sr_3_Ir_2_O_7_ single crystals, Sr_3_(Ir_1−*x*_Mn_*x*_)_2_O_7_ with *x* = 0, 0.09, 0.18, 0.36 by means of infrared spectroscopy. The substitution of Mn ions, Mn^3+^ (3*d*^4^) or Mn^4+^ (3*d*^5^), is expected to dope holes to the system and to dilute the SOC, which can induce an insulator–metal transition. Upon Mn doping, the optical excitations between the *J*_eff_ bands were suppressed and a low-energy in-gap excitation at the energies $\hslash \omega \le$ 0.25 eV appeared. The low-energy in-gap excitation reflects hole doping and is universally observed in doped iridates^17–20^. While the in-gap excitation was enhanced with Mn doping, it did not evolve into the coherent Drude-like response in Sr_3_(Ir_1−*x*_Mn_*x*_)_2_O_7_, which is in sharp contrast to other doped iridates. In addition, the resonance energies of the optical excitations between the *J*_eff_ bands hardly changes with Mn doping, indicating the stiffness of the SOC. At $\hslash \omega \ge$ 1 eV, a distinct optical excitation which has not been observed in 4*d* transition-metal doped iridates, such as Sr_2_(Ir,Rh)O_4_ and Sr_n+1_(Ir,Ru)_n_O_3n+1_, emerged with Mn doping. The resonance energy of this optical excitation is higher than those of the transitions between *J*_eff_ bands. The observation of the high-energy optical transition suggests that the on-site energy of Mn ions is quite different from that of Ir ions. The large difference in the on-site energies can lead to a strong disorder and a resilience of the SOC and thus can be associated with the absence of the insulator–metal transition in Sr_3_(Ir_1−*x*_Mn_*x*_)_2_O_7_. Mn doping also alters the temperature evolution of the electronic response. The strong anomaly in the optical response of the parent compound at the antiferromagnetic transition temperature *T*_N_ disappeared upon Mn doping, which may be associated with charge disproportionation of the Ir ions.

## Results and discussion

### Infrared-active phonon modes

**Figure 1:**
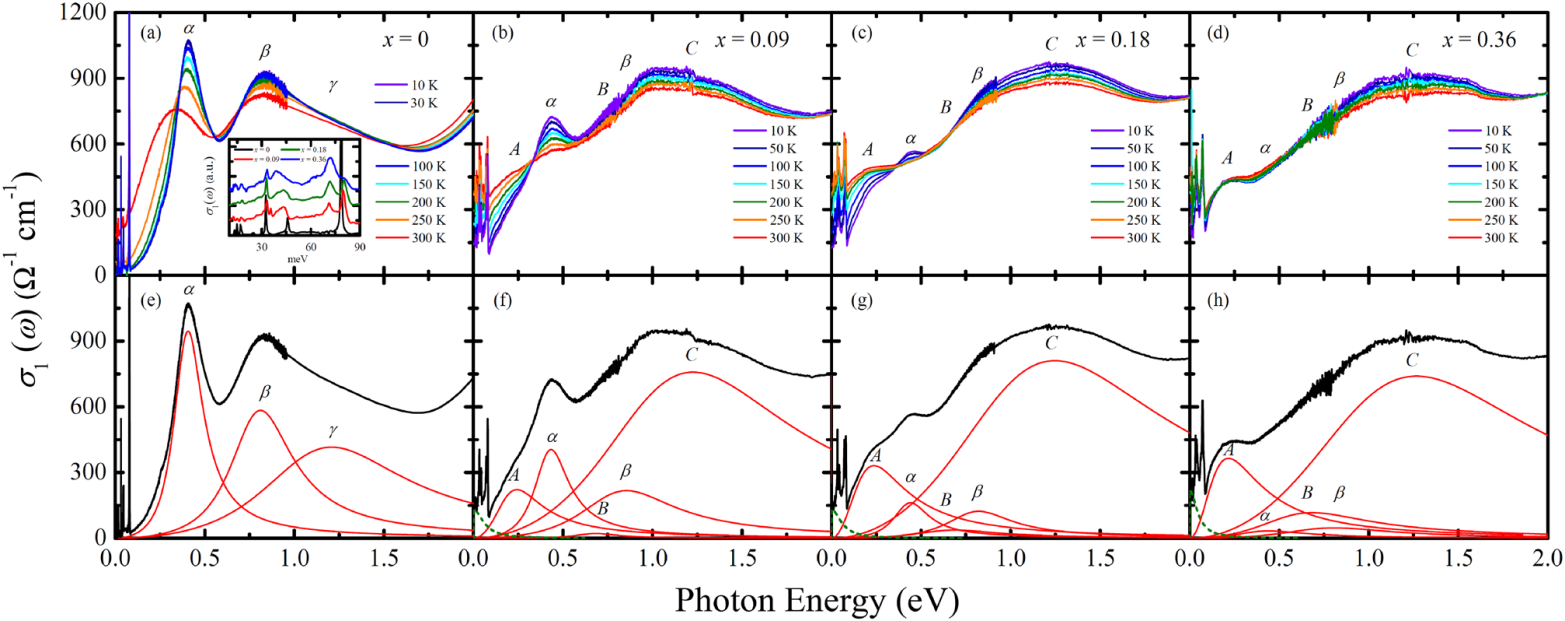
Temperature dependent optical conductivity spectra *σ*_1_(*ω*) of Sr_3_(Ir_1-*x*_Mn_*x*_)_2_O_7_ for (**a**) *x* = 0, (**b**) *x* = 0.09, (**c**) *x* = 0.18, (**d**) *x* = 0.36 from *T* = 10 to 300 K. The inset of (**a**) displays the *σ*_1_(*ω*) below 100 meV. The baseline of each spectrum is shifted for clarity. Fitting results of the Drude-Lorentz oscillator model (dashed lines: Drude oscillators, solid lines: Lorentz oscillators) for (**e**) *x* = 0, (**f**) *x* = 0.09, (**g**) *x* = 0.18, (**h**) *x* = 0.36 at *T* = 10 K.

The inset of Fig. 1a shows the optical conductivity data at 10 K in the energy region below 100 meV where the infrared-active phonon modes are observed. For the parent compound, the six phonon modes expected for the layered perovskite structure are registered: three external modes below 20 meV, two bending modes at 32 and 46 meV, and a stretching mode at 78 meV^21^. Upon 9% Mn substitution, the stretching mode (78 meV) and one of the bending modes (46 meV) of the parent compound become weaker and broader, and new modes appear at about 41 and 71 meV. The former and latter energies are close to the resonance energies of the bending mode and the stretching mode of the parent compound, respectively. This suggests that the modes at 41 and 71 meV can be attributed to the bending and the stretching modes associated with the Mn–O bonds. Further Mn substitution induces gradual suppression and enhancement of the modes related to the Ir–O and Mn–O bonds, respectively, which are demonstrated most clearly in the stretching modes. Further Mn substitution leads to gradual suppression and enhancement of the phonon modes associated with the Ir–O and Mn–O bonds, respectively. This observation indicates a continuous evolution of the lattice properties with Mn substitution, which is consistent with the results of the previous neutron diffraction measurements^22^.

An exception to the gradual evolutions of the phonon modes is observed in the *x* = 0.09. A mode at 35 meV is observed only in the *x* = 0.09 compound. The resonant elastic X-ray scattering (REXS) data showed the pronounced splitting of the nuclear Bragg reflections of the *x* = 0.09 in the antiferromagnetic phase^22^. The neutron diffraction data also revealed an enhancement of the monoclinic distortion in the compounds with low-level of Mn doping (*x* = 0.11)^22^. At higher Mn concentrations, the enhancement of the monoclinic distortion disappeared, which was attributed to disorder due to increased Mn substitution or the disappearance of the antiferromagnetic order^22^. It was suggested that for low-level of Mn substitution where the long-range antiferromagnetic order remains intact a strong magnetostructural coupling may play an important role for the enhancement of the monoclinic distortion^22^. The close similarities between the doping evolutions of the infrared-active phonon modes and the structural anomalies in the REXS and the neutron diffraction data suggest that the magnetostructural coupling may be responsible for the 35 meV mode in the *x* = 0.09 compound.

### Doping evolution of the electronic response

Figure 1 shows the real part of the optical conductivity spectra *σ*_1_(*ω*) of Sr_3_(Ir_1−*x*_Mn_*x*_)_2_O_7_. In the *σ*_1_(*ω*) of the parent compound, two prominent peaks, labeled as *α* and *β* are observed. The peak *α* corresponds to the optical transition from the *J*_eff_ = 1/2 lower Hubbard band (LHB) to the *J*_eff_ = 1/2 upper Hubbard band (UHB). The peak *β* corresponds to the optical transitions from the *J*_eff_ = 3/2 bands to the *J*_eff_ = 1/2 UHB^5,7,23^. We note that the *J*_eff_ = 3/2-to-*J*_eff_ = 1/2 UHB transition is composed of two peaks, i.e., *β* and *γ*, as shown in Fig. 1e. Upon Mn doping, the peaks *α* and *β* are suppressed and an in-gap excitation labeled as *A* appears below about 0.25 eV. In addition to the change in the low-energy response, Mn doping induces an enhancement of *σ*_1_(*ω*) in the energy region between 1 and 2 eV, where in the parent compound the peak *γ*, the optical transition from the *J*_eff_ = 3/2 bands to the *J*_eff_ = 1/2 upper Hubbard band, is located. Since the peak *γ* should be suppressed in the Mn-doped samples, similar to the peaks *α* and *β*, the majority of the spectral weight in the energy region between 1 and 2 eV for the Mn-doped compound could not be attributed to the optical transition between the *J*_eff_ bands but should be assigned as a transition associated with the Mn states. To reflect its distinct nature, we labeled the peak at about 1.2 eV in the Mn-doped samples as *C*.

**Figure 2:**
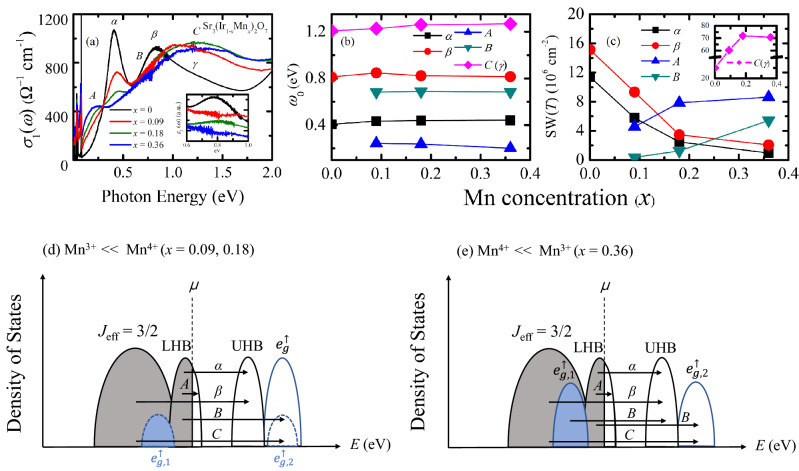
(**a**) Optical conductivity spectra of Sr_3_(Ir_1−*x*_Mn_*x*_)_2_O_7_ at 10 K. The inset shows the imaginary parts of the dielectric constant. The spectra are shifted vertically for clarity. (**b**) Doping dependence of the resonance energies of the peaks *α*, *β, A, B, C* (*γ*) at 10 K. (**c**) Doping dependence of the spectral weights of the peaks *α*, *β*, *A, B, C* (*γ*). The inset shows the doping dependence of the SW of the peak *C*. Schematic band diagram of Sr_3_(Ir_1−*x*_Mn_*x*_)_2_O_7_ with the cases where (**d**) the Mn^4^^+^ and (**e**) Mn^3^^+^ ions dominate. The bands drawn with dashed lines in (**d**) represent the ${e}_{g,1}^{\uparrow }$ and ${e}_{g,2}^{\uparrow }$ states of the minority Mn^3^^+^ ions in the *x* = 0.09 and 0.18 compounds. The LHB (UHB) in (**d**) and (**e**) denotes the *J*_eff_ = 1/2 lower (upper) Hubbard bands.

Optical conductivity data at 10 K in Fig. 2a illustrate the evolution of the electronic structure with Mn doping more clearly. One of the most prominent changes is the drastic suppression of the peak *α*. Part of the spectral weight of the peak *α* is shifted across an isosbestic point at about 0.25 eV to the lower-energy in-gap excitation, peak *A*, which is one of the universal characteristic features of the filling-controlled insulator–metal transition in correlated electron systems^24,25^. For a system exhibiting the filling-controlled insulator–metal transition, charge carrier doping leads to a suppression of the optical transitions across the gap and spectral weight shift to fill the gap with an incoherent in-gap excitations. Upon further doping, a weak coherent Drude-like response appears before a fully coherent Drude-like peak develops with the merger between the coherent and the incoherent responses. These universal behaviors were registered in the optical response of Rh- or Ru-doped Sr_n+1_Ir_n_O_3n+1_ (*n* = 1, 2). Both the Rh and Ru substitution result in hole doping and induces an insulator–metal transition. In the optical conductivity data of Sr_2_(Ir,Rh)O_4_ and Sr_3_(Ir,Ru)_2_O_7_, a clear Drude-like peak appeared upon 5% Rh and 34% Ru doping, respectively^17,19^. In sharp contrast, for Sr_3_(Ir_1-*x*_Mn_*x*_)_2_O_7_, the incoherent in-gap excitation does not evolve into the coherent Drude-like peak but remains incoherent up to the highest Mn concentration of *x* = 0.36, which suggests that the doped holes remain localized.

Anderson localization can be responsible for the incoherent nature of the low-energy optical response^26,27^. Anderson localization is mainly due to disorders in the system. In a correlated Mott system, the cooperation between the randomness and the electronic correlations can induce a soft gap in the electronic density of states^27–31^, thus hindering coherent charge transport. Accordingly, the dc resistivity follows variable-range hopping (VRH)^32^. The resistivity data of Sr_3_(Ir_1−*x*_Mn_*x*_)_2_O_7_ indeed display the VRH behavior in a wide range of temperature^22^. We note that the VRH behavior of the dc resistivity was also registered in insulating/bad metallic Rh- and Tb-doped Sr_2_IrO_4_ compounds^33,34^.

In order to obtain more quantitative information on the evolution of the electronic structure with Mn doping, we analyzed the *σ*_1_(*ω*) using Drude-Lorentz oscillator model:

1 $$\sigma _{1} \left( \omega \right) = ~\frac{1}{{4\pi }}\frac{{\omega _{p}^{2} \gamma _{D} }}{{\omega ^{2} + \gamma _{D}^{2} }} + \mathop \sum \limits_{k} \frac{{S_{k}^{2} }}{{4\pi }}\frac{{\omega ^{2} \gamma _{k} }}{{\left( {\omega ^{2} - \omega _{{0,k}}^{2} } \right)^{2} + \omega ^{2} \gamma _{k} }}$$

*ω*_p_ and *γ*_*D*_ are the plasma frequency and the scattering rate of the Drude oscillator, respectively. *S*_*k*_, *γ*_*k*_, and *ω*_0,*k*_ are the strength, the width, and the resonant frequency of the Lorentz oscillator, respectively. The result of the Drude-Lorentz oscillator model fit for the 10 K data of Sr_3_(Ir_1−*x*_Mn_*x*_)_2_O_7_ is displayed in Fig. 1e–h. One Drude (*D*) oscillator, three (*α*, *β*, *γ*) and five (*α*, *β*, *A*, *B*, *C*) Lorentz oscillators are needed to reproduce the *σ*_1_(*ω*) data of the parent and Mn-doped compounds, respectively. The peak *A* represents the in-gap excitation. The peak *B* is required to account for the little change in *σ*_1_(*ω*) at about 0.6–0.7 eV despite the suppression of the peak *β*. The parameters extracted from the fitting are summarized in Fig. 2b and c.

The Lorentz oscillator model analysis provides important information on the evolution of the electronic structure of Sr_3_(Ir_1−*x*_Mn_*x*_)_2_O_7_. We note that the resonance energies of the peak *α* and *β* hardly change with Mn doping, as shown in Fig. 2b. It should be mentioned that, whereas the resonance energy of the peak *β* is not clearly resolved in *σ*_1_(*ω*) due to its overlap with the neighboring optical transitions, it can be identified in the imaginary part of the dielectric function *ε*_2_(*ω*) [inset of Fig. 2a]. Since the separation of the peaks *α* and *β* is proportional to the magnitude of the SOC, *λ*_SO_, this observation indicates that the SOC is resilient against Mn doping. The robustness of the SOC in the Mn-doped compounds samples is further supported by the large branching ratio from a recent x-ray absorption spectroscopy study^22^.

A recent ARPES study on Sr_2_(Ir,Ru)O_4_ and Sr_2_(Ir,Rh)O_4_ suggested that the reduction of the SOC is strongly dependent on the impurity potential^13^. It was shown that the large difference between the on-site energies of the host and impurity states can prevent their hybridization and thus the dilution of the SOC. Our observation of the robustness of the SOC as well as the incoherent charge transport of Sr_3_(Ir_1−*x*_Mn_*x*_)_2_O_7_ suggests that the Mn states may be located away from the Ir states in energy.

**Figure 3:**
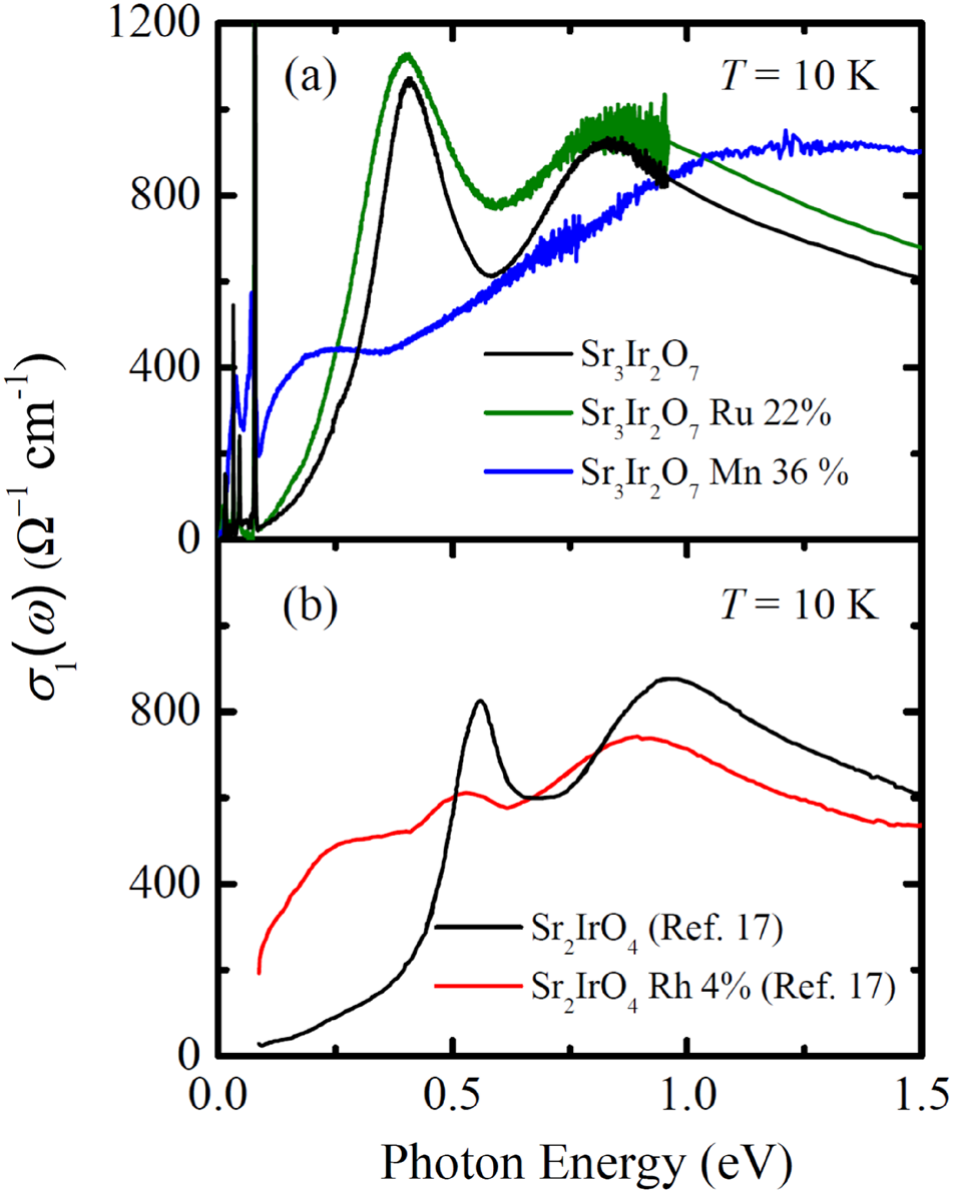
Optical conductivity spectra of (**a**) Sr_3_Ir_2_O_7_, Sr_3_(Ir_0.78_Ru_0.22_)_2_O_7_, Sr_3_(Ir_0.64_Mn_0.36_)_2_O_7_ and (**b**) Sr_2_IrO_4_, Sr_2_Ir_0.96_Rh_0.04_O_4_. The data of Sr_3_(Ir_0.78_Ru_0.22_)_2_O_7_ and Sr_2_IrO_4_/Sr_2_Ir_0.96_Rh_0.04_O_4_ are from refs. 19 and 17, respectively.

The strong enhancement of *σ*_1_(*ω*) in the energy region between 1 and 1.5 eV, which leads to the development of the peak *C*, with Mn doping indicates that the Mn states are indeed located away from the Ir *J*_eff_ = 1/2 states in energy: the resonance energy of the peak *C* is larger than those of the optical transitions between the *J*_eff_ =1/2 bands. In Sr_3_(Ir,Ru)_2_O_7_ and Sr_2_(Ir,Rh)O_4_ where the Ru or Rh states were closer in energy to the Ir *J*_eff_ = 1/2 bands^13,14^ than the Mn states, Ru or Rh substitution do not induce the enhancement of *σ*_1_(*ω*) in the energy region between 1 and 1.5 eV^17–19,35^, as shown in Fig. 3a and b.

The assignment of the peak *C* should depend on the valence state of Mn ions^36–38^. In perovskite manganites, Mn ions can have 4 + (3*d*^3^) or 3 + (3*d*^4^) valence state. For Mn^4+^ ions, the doubly-degenerate *e*_g_ spin-up (${e}_{g}^{\uparrow }$) bands are closest to the Fermi level: it is located above the Fermi level by about 1.71 eV in Sr_3_Mn_2_O_7_^39^. For Mn^3+^ ions, the degeneracy of the ${e}_{g}^{\uparrow }$ bands is lifted by Jahn–Teller effects and the split ${e}_{g,1}^{\uparrow }$ and ${e}_{g,2}^{\uparrow }$ bands are located above and below the Fermi level by at least about 0.6 eV^40^.

Magnetization measurements of Sr_3_(Ir_1−*x*_Mn_*x*_)_2_O_7_ suggested a shift in the Mn valence from 4+ to 3+ at *x* $\approx$ 0.25^22^. However, a comparison between the low-energy optical response of Sr_3_(Ir_1−*x*_Mn_*x*_)_2_O_7_ and Sr_3_(Ir,Ru)_2_O_7_/Sr_2_(Ir,Rh)O_4_ suggests that Mn^3+^ ions should exist in *x* = 0.09 and 0.18 samples. As shown in Fig. 3a, the conductivity data of Sr_3_(Ir,Ru)_2_O_7_ barely changes even upon 22% Ru doping^19^. The peak *α* remains robust against Ru doping and in-gap excitations do not emerge. The sizeable changes in *σ*_1_(*ω*) occur only beyond the critical Ru concentration of about 0.35 at which the transport data indicates an insulator-to-metal transition^41^. This behavior suggests that the charge transfer between Ir^4+^ and Ru^4+^ ions is prohibited, thus protecting 4+ valence of Ir ions^41–43^. In contrast, it is known that Rh substitution induces hole doping on the Ir sites via electron transfer from Ir ions to Rh at low Rh concentrations, introducing Ir^5+^ and Rh^3+^ pairs^44–47^. The corresponding *σ*_1_(*ω*) of Sr_2_(Ir,Rh)O_4_ exhibits the suppression of the peak *α* and the emergence of an in-gap excitation^17^. The close similarities between the optical responses of Sr_3_(Ir_1−x_Mn_*x*_)_2_O_7_ and Sr_2_(Ir,Rh)O_4_ suggest that Mn substitution changes the filling of the *J*_eff_ = 1/2 LHB via hole doping and induces the formation of Mn^3+^ and Ir^5+^ ions. Therefore, we suggest that Mn^3+^ ions coexist with majority Mn^4+^ ions at low Mn concentrations and the portion of Mn^3+^ ions increases with increasing Mn doping. Based on the inference of the valence of Mn ions from the magnetization measurements^22^ and our optical data, the peak *C* can be assigned mainly as a transition from *J*_eff_ = 3/2 state to Mn ${e}_{g}^{\uparrow }$ state for *x* = 0.09 and 0.18 and a transition from *J*_eff_ = 3/2 to Mn ${e}_{g,2}^{\uparrow }$ state for *x* = 0.36 compound. Then, the peak *B* at about 0.68 eV can be assigned as a transition from *J*_eff_ = 1/2 LHB to Mn ${e}_{g}^{\uparrow }$ or ${e}_{g,2}^{\uparrow }$ state. The corresponding schematic diagrams of the electronic density of states are displayed in Fig. 2d and e.

The variation of the SW of the peaks *B* and *C* with Mn doping supports the assignments. As shown in Fig. 2c, the peak *C* is enhanced with Mn doping up to *x* = 0.18 but is suppressed with further Mn doping. The peak *B* exhibits a rapid enhancement as *x* changes from 0.18 to 0.36. When the valence of Mn ions changes from 4+ to 3+, the Mn ${e}_{g}^{\uparrow }$ state can split into ${e}_{g,1}^{\uparrow }$ and ${e}_{g,2}^{\uparrow }$ due to the Jahn–Teller effect, resulting in the decrease in the density of states of the ${e}_{g}^{\uparrow }$ state. Therefore, the SW of the peak *C*, a transition from the *J*_eff_ = 3/2 state to Mn ${e}_{g}^{\uparrow }$ state should be suppressed. In order to account for the enhancement of the peak *B*, the split ${e}_{g,1}^{\uparrow }$ state should be located between the *J*_eff_ = 3/2 and the *J*_eff_ = 1/2 LHB, so that an optical transition from the Mn ${e}_{g,1}^{\uparrow }$ state to the *J*_eff_ = 1/2 UHB contributes to the spectral weight at the energies where the peak *B* is located. Further studies are desired to investigate the effects of the Jahn–Teller splitting of Mn ${e}_{g}^{\uparrow }$ states.

### Temperature evolution of the optical response

**Figure 4:**
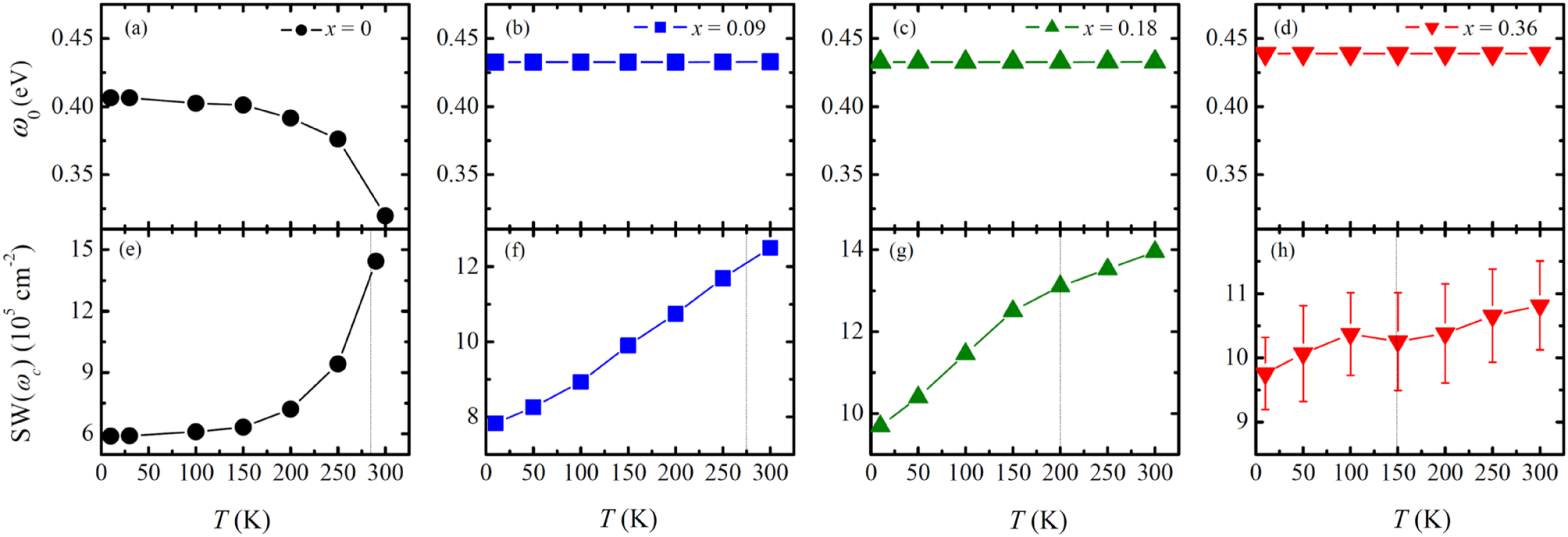
Temperature dependence of the peak *α* of Sr_3_(Ir_1−*x*_Mn_*x*_)_2_O_7_ with (**a**) *x* = 0, (**b**) *x* = 0.09, (**c**) *x* = 0.18, (**d**) *x* = 0.36. Temperature dependence of the SW(*ω*_c_) for (**e**) *x* = 0 (*ω*_c_ = 0.34 eV), (**f**) *x* = 0.09 (*ω*_c_ = 0.34 eV), (**g**) *x* = 0.18 (*ω*_c_ = 0.36 eV) and (**h**) *x* = 0.36 (*ω*_c_ = 0.33 eV). The dashed lines in (**e**)–(**h**) denote the Néel temperature for each sample.

Having identified the effects of Mn doping to the ground-state electronic structure, we discuss the temperature evolution of the low-energy optical response. In the parent compound, the optical conductivity data show clear anomalies at the antiferromagnetic transition temperature *T*_N_, implying a strong spin-charge coupling^18^. As the temperature increases across *T*_N_, the peak α exhibits an abrupt redshift [Fig. 4a]. In addition, the in-gap spectral weight obtained by the integration of *σ*_1_(*ω*) up to the isosbestic point of *ω*_c_ = 0.35 eV, $SW\left({\omega }_{\mathrm{c}}\right)={\int }_{0}^{{\omega }_{\mathrm{c}}}{\sigma }_{1}\left(\omega \right)d\omega$, is significantly enhanced at *T*_N_ [Fig. 4e]. A combined optical spectroscopy and ARPES study demonstrated that these changes in *σ*_1_(*ω*) were attributed to a magnetically driven band shift toward the Fermi level with the suppression of the antiferromagnetic order^18^.

The temperature evolutions of the low-energy optical responses of the Mn-doped compounds suggest that Mn-doping suppresses the spin-charge coupling. Figure 4b–d show that the resonance energy of the peak *α*, which corresponds to the distance between the *J*_eff_ = 1/2 LHB and UHB, is independent of temperature. This observation indicates that the shift of the *J*_eff_ = 1/2 Hubbard bands in energy due to the antiferromagnetic order does not occur in the Mn-doped compounds. In addition, the in-gap spectral weights SW(*ω*_c_) of Sr_3_(Ir_1−*x*_Mn_*x*_)_2_O_7_ calculated with *ω*_c_ = 0.34 (*x* = 0.09), 0.36 (*x* = 0.18), 0.33 eV (*x* = 0.36) show gradual changes with the variation in temperature without any anomaly [Fig. 4e–h]. In addition to the absence of the anomaly at *T*_N_, the magnitude of the change in the SW decreases with Mn doping. It is worth pointing out that the long-range antiferromagnetic order of the *J*_eff_ = 1/2 pseudospin survives up to *x* = 0.25^22^.

The temperature dependence of the optical response of Sr_3_(Ir_1−*x*_Mn_*x*_)_2_O_7_ should be contrasted to that of Sr_3_(Ir,Ru)_2_O_7_. In the latter system, the anomaly in *σ*_1_(*ω*) at *T*_N_, related to the redshift of the peak *α* and the increase in the SW(*ω*_c_), remained robust in the antiferromagnetic and insulating compounds^18,19^. The clear distinction between the Mn- and Ru-doped compounds may be attributed to the different valence states of the dopant ions. Since Ru ion (4+) is isovalent with Ir ions in Sr_3_(Ir,Ru)_2_O_7_, the holes tend to be localized at the Ru sites^41^. Therefore, the *J*_eff_ = 1/2 pseudospin can remain intact despite a substantial Ru doping, which may be associated with the survival of the long-range antiferromagnetic ordering of the *J*_eff_ = 1/2 moments up to about 70% Ru doping. Conversely, as inferred from our optical data, Mn doping induces formation of Ir^5+^ ions. Thus, the Ir ions near the doped Mn ions can lose their *J*_eff_ = 1/2 pseudospin. This parallels the observation of the rather quick suppression of the antiferromagnetic ordering of the Ir moments in Sr_3_(Ir_1−*x*_Mn_*x*_)_2_O_7_^22^. We conjecture that this charge disproportionation and the resulting loss of the pseudospin may be responsible for the rapid suppression of the spin-charge coupling in Sr_3_(Ir_1−*x*_Mn_*x*_)_2_O_7_.

## Conclusion

We studied the doping and temperature dependence of the optical response of Sr_3_(Ir_1−*x*_Mn_*x*_)_2_O_7_ with 0 ≤ *x* ≤ 0.36. We observed that the Mn substitution resulted in the suppression of the Mott gap excitation and the appearance of an in-gap excitation, which is one of the characteristic features of charge carrier doping. The in-gap excitation did not evolve into the Drude-like peak, but remained incoherent up to the highest Mn concentration, which is possibly due to the Anderson localization. While the optical excitations between the Ir *J*_eff_ bands were suppressed, their resonance energies did not change, indicating the robustness of the SOC. Mn doping induced an enhancement of the optical conductivity for energies above 1 eV, which shows that the on-site energy of the Mn states is quite different from that of the Ir *J*_eff_ = 1/2 bands. The doping evolution of the optical response suggests that the Anderson localization and the robustness of the SOC are likely to be attributed to the large difference between the on-site energies of the Mn and Ir states. The temperature dependence of the electronic response was significantly affected by Mn doping. The anomalies of the optical response at the antiferromagnetic ordering temperature, the energy shift of the optical transition between the *J*_eff_ = 1/2 Hubbard bands and the abrupt change in the SW of the in-gap excitation observed in the parent compound, disappeared in the Mn-doped compounds. We ascribe this phenomenon of the spin-charge decoupling to the charge disproportionation due to Mn substitution, resulting in the loss of *J*_eff_ = 1/2 pseudospin.

## Methods

High-quality single crystals of Sr_3_(Ir_1−*x*_Mn_*x*_)_2_O_7_ with *x* = 0, 0.09, 0.18, 0.36 were grown using a halide flux growth technique. Details of the single crystal growth were described in Ref. 22. We measured near-normal incidence reflectivity spectra *R*(*ω*) in the energy region between 5 meV and 1 eV using Fourier transform infrared spectrometer (Vertex 70v, Bruker). We employed *in-situ* gold overcoating technique to obtain accurate reflectivity data^48^. Complex optical constants in the energy region between 0.74 and 5 eV were obtained using spectroscopic ellipsometer (M-2000, J. A. Woollam Co.). Optical conductivity was calculated from the *R*(*ω*) data through Kramers-Kronig transformation.

## Data Availability

The data used and/or analysed during the current study are available from the corresponding author on reasonable request.
